# Anise Hyssop *Agastache foeniculum* Increases Lifespan, Stress Resistance, and Metabolism by Affecting Free Radical Processes in *Drosophila*

**DOI:** 10.3389/fphys.2020.596729

**Published:** 2020-12-16

**Authors:** Olha M. Strilbytska, Alina Zayachkivska, Alexander Koliada, Fabio Galeotti, Nicola Volpi, Kenneth B. Storey, Alexander Vaiserman, Oleh Lushchak

**Affiliations:** ^1^Department of Biochemistry and Biotechnology, Vasyl Stefanyk Precarpathian National University, Ivano-Frankivsk, Ukraine; ^2^D.F. Chebotarev Institute of Gerontology, National Academy of Medical Sciences (NAMS), Kyiv, Ukraine; ^3^Department of Life Sciences, University of Modena and Reggio Emilia, Modena, Italy; ^4^Department of Biology, Carleton University, Ottawa, ON, Canada

**Keywords:** *Agastache foeniculum*, lifespan, metabolism, oxidative stress, *Drosophila*

## Abstract

Anise hyssop, *Agastache foeniculum*, is a widely used medicinal herb with known antioxidant properties. We studied how dietary supplementation with dried *A. foeniculum* leaf powder affected physiological and metabolic traits as well as activities of antioxidant enzymes and markers of oxidative stress in *Drosophila melanogaster*. Dietary hyssop extended the lifespan in a sex and genotype independent manner over a broad range of concentrations up to 30 mg/ml. Dietary supplementation with the herb significantly increased fecundity, resistance to oxidative stress and starvation. Higher transcript levels of *Drosophila* insulin-like peptide (*dilp2*) and decreased *dilp3* and *dilp6* transcripts together with increased levels of glycogen and triacylglycerols support an alteration of insulin signaling by the plant extract. Increased enzymatic activities of superoxide dismutase and aconitase as well as elevated protein and low molecular mass thiols also supported an alteration of free radical process in flies treated with dietary *A. foeniculum* leaf powder. Thus, physiological and metabolic traits as well as free radical processed may be affected by active compounds detected in extracts of anise hyssop leaves and contribute to the increased lifespan and reproductive (egg-laying) activity observed.

## Introduction

Anti-aging pharmacology is an extremely promising field as it could allow humans to substantially increase lifespan and healthspan (Vaiserman and Lushchak, 2017; Piskovatska et al., 2019). Aging is a normal physiological process that is regulated by a set of genes and signaling pathways that are evolutionarily conserved in eukaryotes. Recent research has concentrated on the influence of different naturally occurring compounds on the lifespan of model organisms. Pathways controlling lifespan and aging are partially conserved in a wide range of species, from yeast to humans (Bitto et al., 2015; Fontana and Partridge, 2015). *Drosophila melanogaster* is emerging as an important model to study anti-aging medications. Since *D. melanogaster* can be easily manipulated genetically and experimentally, it has served as a good model for examining the anti-aging properties of resveratrol (Bass et al., 2007; Wang et al., 2013), 4-phenylbutyrate (Kang et al., 2002), caffeine (Nikitin et al., 2008), curcumin (Lee et al., 2010), statin (Spindler et al., 2012), *Rhodiola rosea* (Gospodaryov et al., 2013), *Rosa damascena* (Schriner et al., 2012), blueberry extract (Peng et al., 2012), and many other natural compounds.

Longevity can be modulated by preventing age-related diseases, including cardiovascular disease, type 2 diabetes, cancer and Alzheimer’s disease. It is well-known that dietary antioxidants play potential roles in the prevention of age-related diseases (Meydani, 2001). Numerous studies have shown that plant extracts from *R. rosea* or *Ludwigia octovalvis* can extend lifespan of more than one model organism. In *Drosophila, R. rosea* delayed an age-related decline of locomotor activity and increased stress resistance (Gospodaryov et al., 2013), whereas *Theobroma cacao* increased lifespan in *D. melanogaster* due to its antioxidant properties (Bahadorani and Hilliker, 2008). Being known for its powerful antioxidant activity, antibacterial and hepatoprotective properties, rosemary extract (*Rosmarinus officinalis* L.) produced a longevity phenotype in *Drosophila* that was associated with increased superoxide dismutase and catalase activities (Wang et al., 2017). Extended lifespan was also observed when fruit flies were fed *L. octovalvis* that is a rich in antioxidants including polyphenolic compounds, phytosterols, and squalene in either regular or high-calorie diets (Lin et al., 2014).

Among herbs and spices, *Agastache foeniculum* (AF), known as anise hyssop, has been given much attention in particular for its high antioxidant activity and is often used for the production of essential oils. Traditional medicine applies AF for acute respiratory diseases, functional disorders of the gastrointestinal tract, and inflammatory diseases of the urinary system (Marcel et al., 2013). Three groups of compounds were identified in the essential oil of AF: monoterpenes (sylvestrene and 1-octen-3-ol acetate), phenylpropenes (methyl chavicol, eugenol, and methyl isoeugenol), and sesquiterpenes (β-caryophyllene, spathulenol, and caryophyllene oxide) (Ivanov et al., 2019). Studies have investigated the antimicrobial and antioxidant activity of hyssop essential oil (Ivanov et al., 2019), as well as antimutagenic, anti-nociceptive, anti-inflammatory and cytotoxic activity with cancer cell lines (Zielińska and Matkowski, 2014). Individual parts of the plant are used for different purposes but leaves are the most useful ones. Indeed, the leaves of hyssop can be used in herbal tea or added fresh in small quantities to a salad with other greens. The dried leaves can be used for medicinal purposes to treat coughs, fevers, wounds, and diarrhea. In this regard, we decided to evaluate the effect of AF on physiology, metabolism and free radical processes in *D. melanogaster*. We found that extracts of plant leaves are full of flavonoids and active compounds.

The results revealed that the lifespan of both sexes and two fly lines (*Canton S* and *w*^1118^) was extended significantly by dietary supplementation with different concentrations of AF dry leaves. Furthermore, AF significantly increased fertility, climbing ability, and resistance to oxidative stress and starvation of *Canton S* flies. Enzymatic activities of aconitase and catalase were also significantly increased when *A. foeniculum* was consumed and *Canton S* flies fed diets with AF added displayed lower body glucose content, but, higher levels of stored glycogen and triglycerides.

## Materials and Methods

### Analysis of Extracts by HPLC/MS

The composition of herbal extracts was analyzed using high-performance liquid chromatography (HPLC) with subsequent mass spectrometry. The high-performance liquid chromatography equipment was from Jasco (Tokyo, Japan) and included a pump (model PU-1580), UV detector (UV-1570), Rheodyne injector equipped with a 20 μL loop, and Jasco-Borwin software (rel. 1.5). Samples were separated using a 250 × 4.6-mm stainless-steel column Discovery-C18 4 μm 80 Ä (from Sigma-Aldrich). The eluates were (A) 0.5% acetic acid and (B) acetonitrile. Separations were performed at room temperature by solvent gradient elution from 0 min at 50% A/50% B to 60 min at 100% B at a flow rate of 0.8 mL/min. A UV detector set at 260 nm was also used on-line with the HPLC equipment. An Agilent 1100 VL series mass spectrometer (Agilent Technologies, Inc., Santa Clara, CA, United States) was further used on-line with the HPLC equipment. The electrospray interface was set in negative ionization mode with a capillary voltage of 3,500 V and a temperature source of 350°C in full scan spectra (200–2,200 Da, 10 full scans/s). Nitrogen was used as a drying (9 L/min) anebulizing gas (11 p.s.i.). Software versions were 4.0 LC/MSD trap control 4.2 and Data Analysis 2.2 (Agilent Technologies, Inc.).

### Antioxidant Properties

Dry crushed leaves of *Agastache foeniculum* were purchased from a local store in Ivano-Frankivsk (Ukraine). Leaves were ground to a powder and an aqueous extraction was performed by adding the powder to hot water (85–90°C) in concentrations of 2.5, 5, 10, or 30 mg/ml. Liquid extracts were filtered and kept at 4°C for 24 h. Extracts were used the next day for experiments. This protocol of extraction was used because it closely matched the conditions for preparing food for fly experiments in which leaf powder was added into hot fly food (85–90°C) that was then poured into vials and kept at 4°C before use. To assess the antioxidant of ability, plant extracts were assessed for their ability to scavenge ABTS^+^ radical cations or reduce ferric ions were determined as described previously (Bayliak et al., 2016).

#### Resistance to Starvation and Oxidative Stress

Flies of *Canton S* strain were fed control or experimental food (5 or 10 mg/ml plant powder) for 30 days. Flies were separated under light CO_2_ anesthesia and kept overnight for recovery. Starvation resistance was measured in flies given only 1% agar as a food source. To study resistance to oxidative stress 25–30 flies of each experimental cohort were transferred into empty vials for 2 h for starvation. After starvation, flies were transferred into vials containing folded and rammed strips (2.4 × 12 cm) of 4-layer cellulose filter paper soaked with 0.8 ml of 20 mM menadione in 5% sucrose solution (Lushchak et al., 2014). Dead flies were counted at 9 a.m., 3 p.m., and 9 p.m. Stress resistance was expressed as the percentage of flies that survived over the time.

### Enzyme Activities

Flies, fed control or herb-supplemented diet for 30 days were fixed by freezing in liquid nitrogen and kept at −80°C before use. For analysis were homogenized using a Potter-Elvejhem glass homogenizer (1:10 w:v) in cold 50 mM potassium phosphate buffer, pH 7.5, containing 0.5 mM EDTA and 1 mM phenylmethylsulfonyl fluoride. Centrifugation was performed at 16,000 g for 15 min at 4°C in an Eppendorf 5415R centrifuge (Germany). The supernatants were collected and used for the determination of enzyme activities.

The activities of superoxide dismutase (SOD) and catalase were measured as described previously (Lozinsky et al., 2012). Briefly, SOD activity was assayed at 406 nm by inhibition of quercetin oxidation by superoxide anion. One unit of SOD activity was defined as the amount of soluble protein that inhibited the maximum rate of quercetin oxidation by 50%. Catalase activity was determined by the rate of hydrogen peroxide decomposition at 240 nm. Enzyme activity was calculated using an extinction coefficient for hydrogen peroxide of 39.4 M^–1^ cm^–1^.

Aconitase activity was measured as a decrease in substrate concentration as described earlier (Lozinsky et al., 2013). Briefly, the decrease in absorbance at 240 nm was followed for 2 min and the extinction coefficient used for calculations was 3.701 M^–1^ cm^–1^ for cis-aconitate.

All reactions were started by addition of enzyme supernatant. Activities were measured at 25°C and expressed per milligram of soluble protein in the supernatant.

### Protein and Low Molecular Mass Thiols

The content of free thiols was determined by the Ellman’s method using DTNB, as described previously (Lushchak et al., 2011). The content of protein thiols groups was calculated as the difference between total and low molecular mass thiols. The content of protein thiols was expressed as μmol per mg protein and low molecular weight thiols as mg per mg of wet weight.

### Fly Stocks and Rearing

Fruit flies of *Canton S* (BDRC #64349) and *w*^1118^ (BDRC #3605) strains were obtained from Bloomington Stock Center (Bloomington, IN, United States). The flies were cultured in a standard molasses medium (7.5% molasses, 5% yeast, 6% corn, 1% agar, 0.18% methylparaben) in uncrowded (70–100 eggs/vial) conditions at 25°C, 60% humidity and photoperiod 12 L:12 D. *Canton S* flies were used throughout this study whereas flies of the *w*^1118^ strain were used only for lifespan assay.

### Lifespan

Newly eclosed flies were transferred into fresh food and kept for 3 days for mating. Then flies were separated by sex under light CO_2_ anesthesia and kept for another day for recovery. About 150 flies of each strain and sex were gently transferred to 1.5 L demographic cages with an attached plastic vial filled with 5 ml control food or experimental food supplemented with different concentrations of anise hyssop (*A. foeniculum*) dry crushed leaves. The standard medium consisted of 5% of dry yeast, 5% of sucrose, 1.2% agar, and 0.18% methylparaben. Herbal powder was directly added to the experimental food (t = 70°C) in different concentrations (2.5, 5, 10, or 30 mg/ml). Food was changed every second day, and dead flies were removed and recorded. The experiment was run in two biological replicates. To study the effects of different yeast concentrations, diet with 5% yeast was defined as 1×. Yeast content was changed to 0.25% to obtain 0.05× diet, 1% to obtain 0.2× diet and 20% to get 4× diet. All experimental diets were supplemented with 5 or 10 mg/ml of herb powder.

### Fecundity

To determine the impact of anise hyssop on fruit fly fecundity 20 female flies were placed into demographic cage supplemented with food vial. Amount of laid eggs was counted 24 h after fresh food was applied. The measurements were repeated every 3 days up to day 30. Four groups of flies were tested per condition.

### Negative Geotaxis and Heat Shock Resistance

Flies were fed control or herb supplemented diet for 30 days. Negative geotaxis, sensitivity and recovery after heat shock were measured as described in Lushchak et al. (2012).

### Glucose, Trehalose, Glycogen, and TAG

Flies were fed experimental diets for 30 days and that used for measurements of metabolites. Flies were decapitated and centrifuged 5 min at 3,000 g to extract hemolymph (Perkhulyn et al., 2015). Pre-weighted whole flies were homogenized in 50 mM sodium phosphate buffer pH 6.5 (1:10 w/v) and centrifuged. Resulted supernatants were used for the determination of glucose, glycogen and trehalose contents. Measurements were performed using a glucose assay kit (Liquick Cor-Glucose diagnostic kit, Cormay, Poland). Glycogen was converted into glucose by incubation with amyloglucosidase from *Aspergillus niger* at 25°C for 4 h followed by measuring glucose. Trehalose was determined in samples after incubation with porcine trehalase to digest trehalose into glucose. For TAG estimation weighed flies were homogenized in 200 mM PBST (phosphate buffered saline containing 0.05% Triton X100), boiled and centrifuged for 10 min at 13,000 g (Rovenko et al., 2015). TAG levels were measured using a diagnostic kit Liquick Cor-TG (PZ 290 Cormay S.A., Łomianki, Poland) following the manufacturer’s instructions. TAG levels were expressed as milligrams per gram of wet weight (mg/gww).

### Gene Expression

Total RNA from heads or whole flies was extracted using an RNeasy Plus Mini Kit (Qiagen), concentration was measured, and 2 μg of total RNA were into converted cDNA with QuantiTect Reverse Transcription Kit (Qiagen). Expression of genes of interest was measured using an ABI Prism 7000 instrument (Applied Biosystems), a SensiFAST SYBR Hi-ROX Kit, and a QuantiTect SYBR Green PCR Kit (Qiagen) under conditions recommended by the manufacturer. Levels of mRNA were measured in heads (*dilp2*, *3*, and *5*) or whole flies (*dilp6, akh, 4ebp, tobi, pepck, bmm*). Each analytical and standard reaction was performed in three technical replicates. The ΔΔCt method was used with rp49 as the reference gene. All primers were as described earlier (Lushchak et al., 2015).

### Statistical Analysis

Fly lifespans and survival under starvation or oxidative stress were compared by a Log Rank test. Activities of antioxidant enzymes, markers of oxidative stress and levels of metabolites were analyzed by a two-way ANOVA followed by a Dunnett’s multiple comparison test. Levels of specific mRNAs were compared by a Student’s *t*-test. Significant differences between groups were accepted by a *p*-value < 0.05. Statistical analysis was performed in Prism Graphpad 5 (GraphPad Software, San Diego, CA, United States).

## Results

### Active Compounds in Extract of *Agastache foeniculum*

Analysis of *A. foeniculum* extract with HPLC-MS identified the presence of many bioactive species previously described to possess beneficial properties. We detected acacetin, apigenin, 3-O-caffeoylquinic acid, calycosin, caffeic acid, 6,7-Dimethoxyquercetin 3-O-glucopyranoside, genistein, kaempferol 3-O-glucoside, rosmarinic acid, tilianin, ursolic acid, and β-sitosterol in significant amounts (Supplementary Table S1). These active compounds have been previously shown to increase the life- and healthspan of different model organisms including *Drosophila*. Moreover, varied additional benefits were described in relation to protection against oxidative stress.

### Antioxidant Properties of *A. foeniculum* Extract and Fly Resistance to Oxidative Stress

**FIGURE 1:**
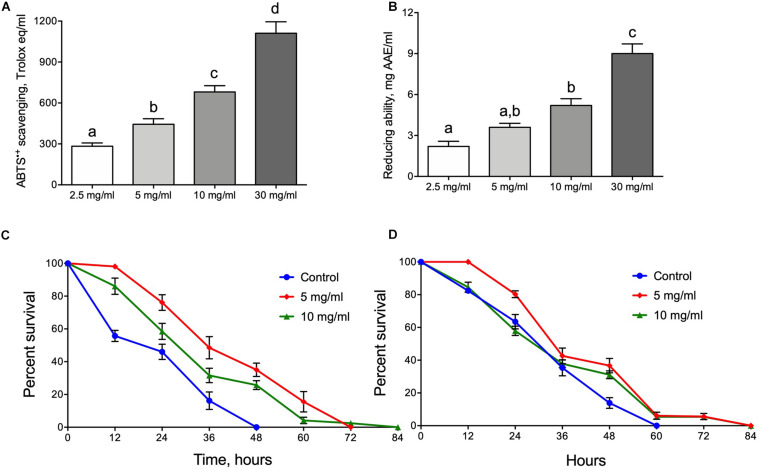
Antioxidant and protective properties of Hyssop extracts. ABTS^+^ radical scavenging activity **(A)** and ability to reduce ferric ions **(B)**. Resistance of male **(C)** and female **(D)** flies to oxidative stress induced by menadione. Data shown in **(A,B)** represent mean ± SEM for four independent extractions. Different letters on **(A,B)** represent groups significantly different from each other (*p* < 0.05).

The antioxidant properties of water extracts of *A. foeniculum* leaves were tested for their ability to scavenge ABTS^+^ radical cations or reduce ferric ions. An extract made of the herb at a concentration of 2.5 mg/ml had scavenging activity of ∼300 Trolox equivalents (Figure 1A). A further increase in herb concentration during extraction did not linearly increase the ability to scavenge ABTS cation. The activity was about 50% higher for the 5 mg/ml extract. Further increases of herb content to 10 and 30 mg/ml increased scavenging ability by approximately 2.2- and 3.3-fold (Figure 1A). A similar tendency was observed when the extracts were tested for the ability to reduce ferric ions (Figure 1B). Reducing ability was about 2.5 mg AAE/ml for the extract made with 2.5 mg/ml herb powder, whereas a 12-fold increase in herb content (30 mg/ml) increased the reduction ability by about 4-fold.

Since the AF extracts were identified as having significant antioxidant activities it was logical to predict that flies fed diets supplemented with AF powder would be more resistant to oxidative stress induced by the redox cycling agent menadione. Figure 1C (male) and Figure 1D (female) show that flies fed diets with AF leaf powder were lived longer under oxidative stress exposure. Male flies fed diets with 5 or 10 mg/ml of AF powder were more resistant by 40 and 75%, respectively (Log Rank test, *p* < 0.0001) (Figure 1C and Supplementary Table S2). AF powder in concentration of 5 mg/ml increased female resistance by 20% as compared to control (Log Rank test, *p* < 0.0001) (Figure 1D and Supplementary Table S2).

### Antioxidant Enzymes, Aconitase, and Markers of Oxidative Stress

**FIGURE 2:**
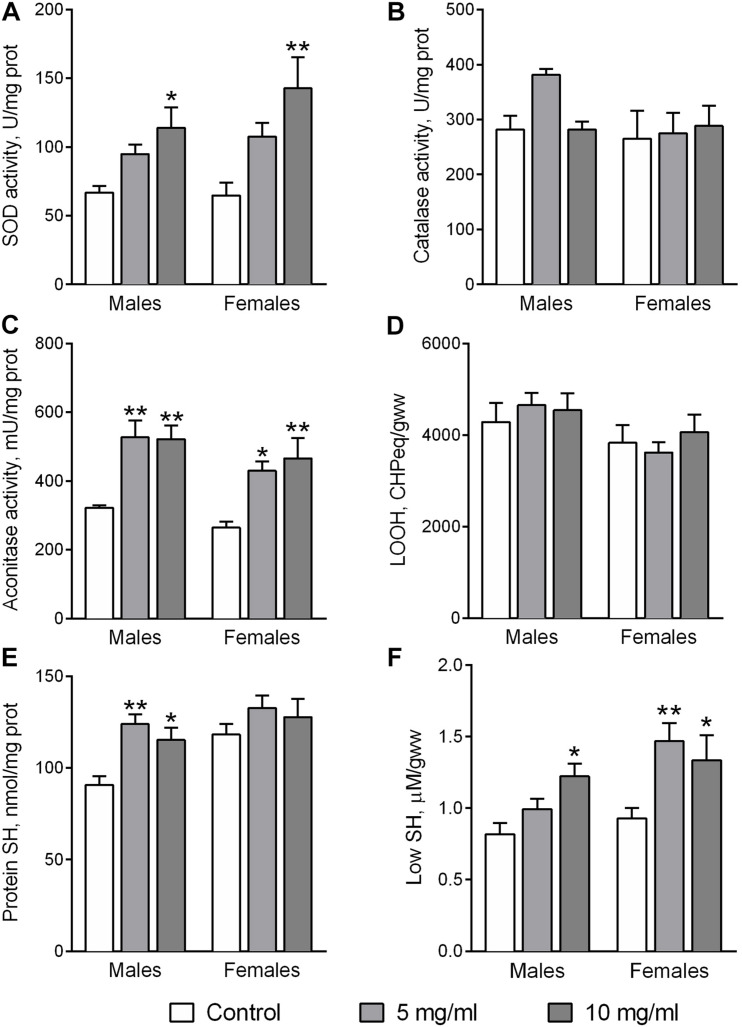
Activities of antioxidant enzymes SOD **(A)**, catalase **(B)**, aconitase **(C)**, and the levels of lipid peroxides **(D)**, protein **(E)**, and low molecular weight thiols **(F)** in flies consuming control diet or diet supplemented with 5 or 10 mg/ml *A. foeniculum*. Results are presented as mean ± SEM of four replicates per group. Group comparisons were performed using Dunnett’s test. Asterisks indicate significant difference from the control flies (*p* < 0.05).

The antioxidant enzymes superoxide dismutase (SOD) and catalase are involved in the detoxification of the reactive oxygen species (ROS) superoxide anion and hydrogen peroxide, respectively. The activity of SOD was affected by food supplementation with AF powder (Figure 2A). Fly treatment with AF supplemented food for 30 days at a concentration of 10 mg/ml significantly increased SOD activity in flies by 1.71-fold in males (Dunnett’s test, *p* = 0.014) and 2.21-fold in females (Dunnett’s test, *p* = 0.010). However, dietary AF did not influence the activity of catalase (Figure 2B).

Increased ROS production and/or a decrease of the detoxification potential can induce oxidative stress (Lushchak, 2014). This may be reflected by specific markers such as the activity of the ROS-sensitive enzyme aconitase, protein carbonyl groups, lipid peroxides, protein and low molecular mass thiol groups (Lushchak et al., 2011). A higher activity of aconitase was observed in flies of both sexes fed by diets supplemented with 5 or 10 mg/ml AF powder (Figure 2C). Activity increased by about 1.62-fold (Dunnett’s test, *p* = 0.003) and 1.63-fold (Dunnett’s test, *p* = 0.03) in male and female flies, respectively. Interestingly, fly feeding with an AF supplemented diet did not affect the contents of lipid peroxides (LOOH) (Figure 2D) and protein carbonyl groups (not shown). However, the antioxidant properties of AF in flies were reflected in reduced protein and low molecular weight thiol (Low SH) groups (Figures 2E,F). Higher contents of protein SH groups were observed in male flies fed diets with AF in concentrations of 5 mg/ml (Dunnett’s test, *p* = 0.005) and 10 mg/ml (*p* = 0.024). Low SH contents, that are mostly represented by glutathione and cysteine, were significantly higher in flies fed diets with AF (Figure 2F). Diet supplementation with AF increased Low SH by 50% (Dunnett’s test, *p* = 0.011) and 45% (*p* = 0.05) in male and female flies, respectively (Figure 2F).

### Lifespan of Different Strains

**FIGURE 3:**
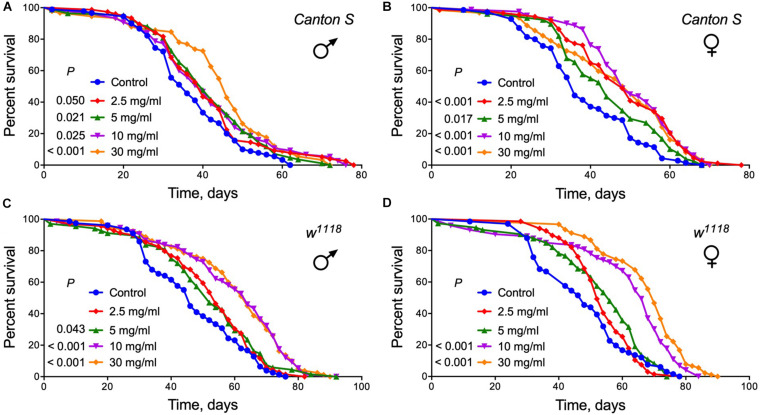
Survival curves for flies of two different lines fed control diet vs. diets supplemented with *A. foeniculum* extract at four different concentrations. **(A)** males of *Canton S*; **(B)** females of *Canton S*; **(C)** males of *w*^1118^; **(D)** females of *w*^1118^. Each curve represents the percentage of alive flies within respect time. The cohorts were compared using a Log rank test (see Supplementary Table S2 for complete statistics).

Identification of nutritional supplements that can delay aging and extend lifespan is one of the most promising ways to improve the quality of life. To directly test whether *A. foeniculum* elicits beneficial effects on lifespan, we raised fruit flies on diets supplemented with of dried crushed leaves added to the food medium at different concentrations. The median lifespan control group of male *Canton S* flies lived approximately 34 days. Lifespan was significantly increased by 15% in male *Drosophila* line *Canton S* fed diets with 5, 10 mg/ml of AF powder or by 32% at 30 mg/ml (Log Rank test, *p* < 0.03) (Figure 3A and Supplementary Table S3). Female flies of the *Canton S* strain lived longer by 37, 33, 37, and 37% when fed diet with 2.5, 5, 10, and 30 mg/ml AF powder, respectively (Log Rank test, *p* < 0.02) (Figure 3B and Supplementary Table S3).

Dietary supplementation with *A. foeniculum* herb also extended the lifespan of male and female flies of another *Drosophila* strain, *w*^1118^ flies. Male flies that consumed foods with 2.5, 5, 10, or 30 mg/ml AF powder lived longer as compared to controls (Log Rank test, *p* < 0.05) (Figure 3C and Supplementary Table S3). We also observed longer lifespan in females, when the herb was added to the food at concentrations of 10 mg/ml (by 40%), 30 mg/ml (by 49%) (Log Rank test, *p* < 0.0001) (Figure 3D and Supplementary Table S3).

### Lifespan on Diets With Different Yeast Contents

**FIGURE 4:**
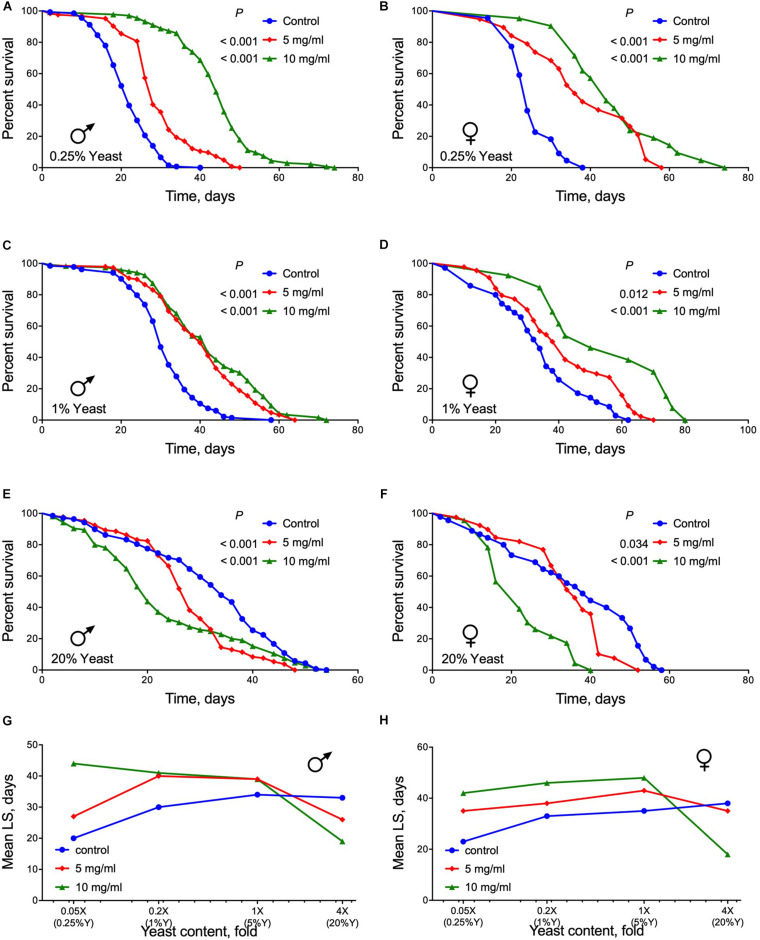
Lifespan of flies fed diets with different concentrations of yeast and supplemented with AF in concentrations of 5 or 10 mg/ml. **(A,B)** show data for yeast concentration 0.05×, **(C,D)** 0.2×, **(E,F)** 4×. Data for male flies are shown in **(A,C,E)** and females in **(B,D,F)**. **(G,H)** show mean lifespan values for male and female flies, respectively.

Dietary protein content is an important determinant of aging and lifespan that is mostly regulated by activity of TOR signaling pathway (Kapahi et al., 2004, 2017; Soultoukis and Partridge, 2016; Lushchak et al., 2017, 2019). We observed higher survival of flies of both sexes when reared on media with 0.05× yeast content and AF powder at both 5 or 10 mg/ml concentrations (Log Rank test, *p* ≤ 0.0001) (Figures 4A,B and Supplementary Table S4). Males and females also lived longer on 0.2× yeast (1%) containing medium with *A. foeniculum* (Log Rank test, *p* < 0.02) (Figures 4C,D and Supplementary Table S4). However, at a very high yeast content (4× or 20%) in conjunction with *A. foeniculum* survival was substantially lower compared with controls (Log Rank test, *p* < 0.04) (Figures 4E,F and Supplementary Table S4). Consequently, we detected the highest mean lifespan of both sexes at 10 mg/ml of *A. foeniculum* under 0.05×, 0.2×, and 1× yeast concentrations in the diet but the lowest mean lifespan occurred under 4× yeast content (Figures 4G,H).

### Fecundity and Negative Geotaxis

**FIGURE 5:**
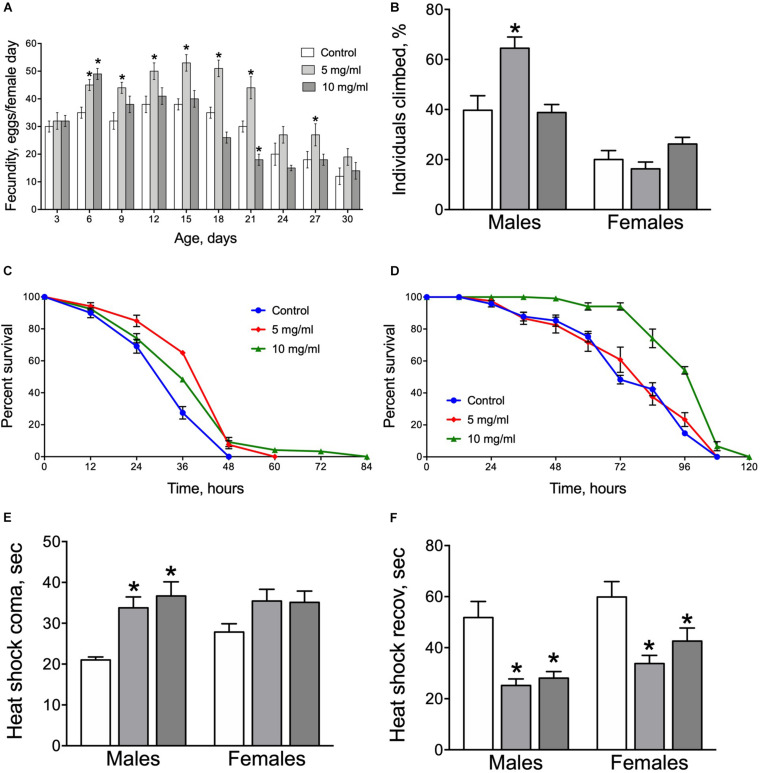
Fecundity **(A)**, negative geotaxis **(B)**, resistance to starvation **(C,D)** and resistance to heat stress **(E)** and recovery **(F)** in flies fed diets supplemented with *A. foeniculum* leaf powder. Data are presented as mean ± SEM with 10 flies tested per cohort. Group comparisons were performed using Dunnett’s test. Asterisks indicates a significant difference from control flies (*p* < 0.05). Color coding for figures **(B,E,F)** as on **(A)**.

Fertility and negative geotaxis are very important life history traits and are excellent indicators of overall health (Flatt, 2020). Early after eclosion, egg production rose in all three groups of adult flies tested (control, 5 and 10 mg/ml) with highest levels after about 12–15 days and a subsequent gradual decrease thereafter (Figure 5A). Flies fed with diets containing *A. foeniculum* (both 5 and 10 mg/ml) had significantly higher daily egg production on day 6 than controls but thereafter fecundity of the 10 mg/ml diet group dropped back by 21 days to at or below control levels. However, fecundity of flies on the 5 mg/ml diet remained significantly higher than controls from day 6 to 27. Consequently, AF has a positive impact on reproduction rate with more obvious effects at moderate concentration (5 mg/ml).

We observed that AF supplementation improved negative geotaxis response only in male flies (Figure 5B). Males that consumed a diet with 5 mg/ml *A. foeniculum* showed approximately 60% higher peroformance as compared to the control group (Dunnett’s test, *p* = 0.008).

### Resistance to Starvation and Heat Stress

To further explore beneficial effects of *A. foeniculum* on lifespan, we assessed effects of this supplement on stress resistance by examining responses of flies to experimental starvation conditions and high temperature. Flies fed diets with AF powder for 30 days demonstrated a longer lifespan under complete starvation conditions with significantly higher survival of male flies fed diets with of 5 mg/ml and 10 mg/ml AF concentrations (Log Rank test, *p* ≤ 0.0005) (Figure 5C). Moreover, *A. foeniculum* supplementation to the medium led to higher starvation resistance in females fed by 10 mg/ml by 37% as compared to control (Log Rank test, *p* = 0.003) (Figure 5D). These data may suggest high nutritional value of the *A. foeniculum*.

We also examined the effect of *A. foeniculum* on the fly resistance to high temperature. Males, that consumed media supplemented with AF powder resisted heat shock significantly longer as compared to controls (Figure 5E). Moreover, the recovery time of flies of both sexes was shorter for the AF consuming groups (Figure 5F). Consequently, *A. foeniculum* in the diet enhances *Drosophila* resistance to the heat stress.

### Metabolites

**FIGURE 6:**
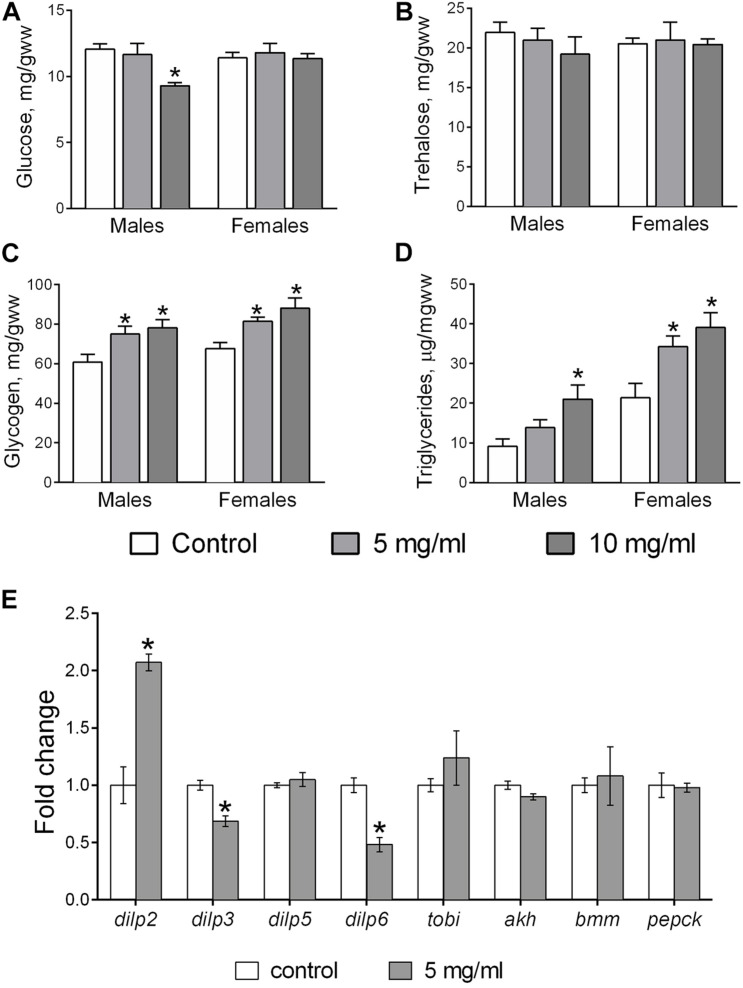
Content of metabolites and mRNA levels for genes involved in regulation of metabolism. Whole body glucose **(A)**, trehalose **(B)**, glycogen **(C)**, TAG **(D)**, and mRNA levels for eight metabolic genes **(E)** in flies treated with AF. Results are mean ± SEM of 4–5 replicates per group. Group comparisons were performed using Dunnett’s test for metabolites and Student’s *t*-test for mRNA. Asterisk indicates significant difference from the control flies with *p* < 0.05.

Glucose, trehalose, glycogen, and TAGs are parameters extensively used for analysis of carbohydrate and fat metabolism in *Drosophila* (Tennessen et al., 2014). We found that consumption of food with 10 mg/ml AF powder decreased the amount of body glucose in males by 17% (Dunnett’s test, *p* = 0.037), but had no impact on females (Figure 6A). Glycogen levels in flies that consumed food with AF in concentrations of 5 and 10 mg/ml was higher by 25 and 30% in males and by 21 and 29% in females, respectively (Figure 6C). TAG concentration was 43% higher in males (at 10 mg/ml AF supplementation) and by 63 and 55% in females fed diets with 5 and 10 mg/ml AF, respectively (Figure 6D). AF supplementation did not affect body trehalose levels of either sex (Figure 6B).

### Gene Expression

We measured the relative expression of eight genes involved in the regulation of intermediary metabolism. The *Drosophila* insulin-like proteins, *dilp2, 3*, and *5*, are co-expressed in insulin producing cells (IPCs) of the brain and hence, their transcript levels were measured in fly heads. *Dilp6* is primarily expressed by fat body cells and its expression and the mRNA levels of adipokinetic hormone (*akh*), target of brain insulin (*tobi*), phosphoenolcarboxykinase (*pepck*), and Brummer, a triglyceride lipase (*bmm*) were measured in whole fly body. Flies that consumed food supplemented with 5 mg/ml AF powder had significantly higher levels of *dilp2* transcripts by about 2-fold in fly heads (Figure 6E; Student’s *t*-test, *p* = 0.004). Moreover, we observed a decrease of mRNA levels for *dilp3* (by 30%; Student’s *t*-test, *p* = 0.005) and *dilp6* (by 50%; Student’s *t*-test, *p* = 0.003) in flies that consumed diet with *A. foeniculum* powder. No changes were observed in the mRNA levels of *dilp5, akh, tobi, pepck*, and *bmm*.

## Discussion

Plant-derived active compounds can be used successfully to treat age-related diseases and extend life- and healthspan (Vaiserman and Lushchak, 2017). Most of the detected components in AF leaf extracts were flavonoids that are intensively studied natural compounds with antioxidant, antineoplastic, antihyperglycemic, cardioprotective, or neuroprotective properties. The HPLC-MS analysis revealed the presence of 24 bioactive compounds as shown in Supplementary Table S1. For example, 2,5-dihydroxycinnamic acid was previously shown to induce apoptosis and may impair autophagic flux in RCC4 renal cancer cells (Selka et al., 2019), whereas 3-O-caffeoylquinic acid acts as a metformin mimetic in extending *Drosophila* lifespan (Liu et al., 2013) and is also widely known for its anti-hyperlipidemic effect (Liu et al., 2013). 6,7-Dimethoxyquercetin 3-O-glucopyranoside has antiradical activity (de Amorim et al., 2013). The flavonoid, acacetin, is a potent inhibitory constituent and affects eclosion rate, feeding, climbing and lifespan in *Drosophila* (Wang et al., 2015). It is often used in humans for Alzheimer’s disease treatments. Flies that were reared on apigenin in their diet showed an increase in lifespan, glutathione, and dopamine content, as well as reduced oxidative stress and apoptosis in a transgenic *Drosophila* model of Parkinson’s disease (Siddique and Jyoti, 2017). Treatment with caffeic acid alleviates oxidative stress induced neurotoxicity in cells and *Drosophila* models (Wu et al., 2017). The naturally occurring calycosin is a known antioxidant that prevents redox imbalance in organisms. It also promotes lifespan in the nematode, *Caenorhabditis elegans*, because of its antioxidant action as well as its ability to enhance stress resistance and reduce ROS through insulin signaling pathway inhibition (Lu et al., 2017). The possible lifespan-extending effects of genistein were previously investigated using *C. elegans* (Lee et al., 2015). Protocatechuic acid similarly extended lifespan in *C. elegans* and increased stress resistance associated with its antioxidant properties (Kim et al., 2014). Caffeic acid was shown to increase antioxidant capacity *in vivo* and, by means of a lipofuscin assay, reduce oxidative damage in nematodes, which resulted in increased lifespan (Pietsch et al., 2011). Ursolic acid (UA) is a naturally occurring triterpenoid exhibiting potential antimicrobial, anti-inflammatory and antiobesity activity and it was shown that dietary UA improved health span and lifespan in male *D. melanogaster* (Staats et al., 2019). β-Sitosterol is capable of extending lifespan, likely via activating AMP-activated protein kinase (AMPK) (Lin et al., 2014). Consequently, all the components detected in *A. foeniculum* extracts have been shown to have lifespan extending effects in various animal models.

We supplemented fly food with crushed dried leaves of *A. foeniculum* (AF) at different concentrations and fed *Drosophila* of two lines (*Canton S* and *w*^1118^). We found strong prolongevity activity that was independent of genotype. In addition, AF considerably enhanced the survival rate of flies under both menadione-induced oxidative stress and starvation conditions. Since there is a clear correlation between lifespan-extension and stress resistance (Kenyon, 2010), the protective action against stress may be a positive factor in AF-mediated lifespan extension.

It is also important that factors extending the lifespan have no negative effects on reproduction that reflect healthspan. In this study, we found a higher fecundity rate in AF-fed flies. Although we detected an increase in reproductive ability of flies, previous studies suggested that phytoestrogen exposure negatively affects reproductive health (Jefferson et al., 2012). We also studied functional aging using a locomotion assay. Interestingly, *Agastache* significantly enhanced the mobility of male flies indicating that this herb provides beneficial effects on healthspan as well as lifespan.

The health- and lifespan of *D. melanogaster* are determined by metabolic rate, stress responses and the expression of metabolic genes (Staats et al., 2019). Our results indicate that the health-promoting effects of AF may be caused by changes in metabolism. Indeed, we observed that the prolongevity phenotype is associated with decrease in body glucose levels and increase in stored glycogen and TAG content. The level of circulating and stored metabolites is regulated by DILPs and the glucagon-like peptide AKH (Bharucha et al., 2008). Notably, a significant reduction of mRNA levels for *dilp3* and *dilp6* and increase *dilp2* were observed in response to dietary supplementation with *Agastache*.

To uncover the mechanisms of how *Agastache* protects against oxidative stress, we analyzed antioxidant enzyme activities using fly homogenates from different treatment groups. Our results show that activities of both aconitase and catalase (in males) were significantly increased by AF in the diet, implicating an attenuation of oxidative stress. Additionally, AF-treated flies may also be more resistant to oxidative stress in part because of the various other phenolic compounds in the plant extract that exhibit antioxidant activity.

Low molecular weight (L-SH) and protein thiols are reliable markers of oxidative stress. Flies, fed with AF showed higher antioxidant potential as demonstrated by higher levels of L-SH in females and protein thiols in males, as well as higher aconitase and catalase activities. We suggest that combined together these changes could be responsible for the lower levels of oxidative damage in AF fed flies. The higher pool of L-SH in females might also be associated with a more extensive biosynthetic metabolism related to the need for egg production (Perkhulyn et al., 2017).

In conclusion, our study revealed lifespan extending effects of *Agastache* in *D. melanogaster* linked in part to elevated antioxidant activity and increased stress resistance. The health benefits of polyphenols found in *Agastache* extract are linked to their capacity to directly scavenge free radicals and other nitrogen species (Halliwell, 2007). However, whether dietary supplementation with AF will be beneficial to mammals and ultimately to humans will require further studies.

## Data Availability Statement

The authors acknowledge that the data presented in this study must be deposited and made publicly available in an acceptable repository, prior to publication. Frontiers cannot accept a manuscript that does not adhere to our open data policies.

## Author Contributions

OS: study design, experimental part, data analysis, and writing of drafts. AZ, AK, FG, NV, and AV: experiments and data analysis. KS: initial draft and critical analysis. OL: idea, study design, data analysis, manuscript preparation, and project management. All authors contributed to the article and approved the submitted version.

## Conflict of Interest

The authors declare that the research was conducted in the absence of any commercial or financial relationships that could be construed as a potential conflict of interest.
