# Supplementary material for: Anise Hyssop Agastache foeniculum Increases Lifespan, Stress Resistance, and Metabolism by Affecting Free Radical Processes in Drosophila
Source: Front Physiol. 2020 Dec 16;11:596729. doi: 10.3389/fphys.2020.596729 (PMC7772399; doi:10.3389/fphys.2020.596729)
Supplement: Supplementary file 1 [file Data_Sheet_1.PDF]

Table S1. Identified bioactive species in the *Agastache* extract used in this study.

2,5-Dihydroxycinnamic acid
3-O-Caffeoylquinic acid
6,7-Dimethoxyquercetin 3-O-glucopyranoside
Acacetin
Apigenin
Caffeic acid
Caffeic acid O-hexoside
Calycosin
Calycosin isomer
Calycosin isomer
Calycosin isomer
Emodin O-glucoside
Fraxiresinol hexoside
Genistein
Kaempferol 3-O-glucoside
Methyl hexadecanoate
Phloretin-hexoside
Protocatechuic acid
Rosmarinic acid
Rosmarinic acid isomer
Rosmarinic acid isomer
Tilianin
Ursolic acid
β-Sitosterol

Table S2. Statistical analysis of fly survival under oxidative stress and starvation at three dose levels of AF extract.

		0 mg/ml	5 mg/ml	10 mg/ml
**Males Oxidative stress Time, hours**	N	100	98	112
	Median	20	28	35
	Difference, %		+40	+75
	χ^2		51.3	22.6
	p value		<0.0001	<0.0001
**Females Oxidative stress Time, hours**	N	192	175	175
	Median	30	36	29
	Difference, %		+20	-3
	χ^2		71.1	28.8
	p value		<0.0001	<0.0001
**Males Starvation Time, hours**	N	120	120	120
	Median	30	36	39
	Difference, %		+20	+30
	χ^2		34.9	12.3
	p value		<0.0001	0.0005
**Females Starvation Time, hours**	N	186	190	190
	Median	71	78	97
	Difference, %		+10	+37
	χ^2		78.9	5
	p value		0	0.026

Table S3. Statistical analysis and comparison of survival for flies of different genotypes on control diet or diets supplemented with *A. foeniculum* extract.

		**0 mg/ml**	**2.5 mg/ml**	**5 mg/ml**	**10 mg/ml**	**30 mg/ml**
**Males *Canton S***	N	149	146	149	139	149
	Median	34	39	39	39	45
	Difference, %		+15	+15	+15	+32
	χ2		3.843	5.384	5.039	17.83
	p value		0.0500	0.0203	0.0248	< 0.0001
**Females *Canton S***	N	142	149	148	148	132
	Median	35	48	43	48	48
	Difference, %		+37	+33	+37	+37
	χ2		19.57	5.735	23.67	13.28
	p value		< 0.0001	0.0166	< 0.0001	0.0003
**Males w^{1118}**	N	148	148	143	148	144
	Median	45	53	51	63	63
	Difference, %		+18	+13	+40	+40
	χ2		3.076	4.092	28.80	30.63
	p value		0.0795	0.0431	< 0.0001	< 0.0001
**Females w^{1118}**	N	147	144	149	138	147
	Median	47	52	55	66	70
	Difference, %		+11	+17	+40	+49
	χ2		1.248	2.961	29.36	61.48
	p value		0.2640	0.0853	< 0.0001	< 0.0001

Table S4. Comparison of survival for flies fed on diets composed of three different yeast concentrations and supplemented with *A. foeniculum* extract at three dose levels.

		0 mg/ml	5 mg/ml	10 mg/ml
**Males 0.25Y (0.05X)**	N	136	124	134
	Median	20	27	44
	Difference, %		+40	+120
	χ^2		67.3	254
	p value		< 0.0001	< 0.0001
**Females 0.25Y (0.05X)**	N	122	119	121
	Median	23	35	42
	Difference, %		+58	+83
	χ^2		14.7	35.8
	p value		0.0001	< 0.0001
**Males 1Y (0.2X)**	N	133	148	119
	Median	30	40	41
	Difference, %		+33	+40
	χ^2		58.9	66.2
	p value		<0.0001	<0.0001
**Females 1Y (0.2X)**	N	135	144	133
	Median	33	38	46
	Difference, %		+19	+56
	χ^2		6.27	11.9
	p value		0.0123	0.0006
**Males 20Y (4X)**	N	138	131	135
	Median	33	26	19
	Difference, %		-16	-28
	χ^2		22.1	13.4
	p value		<0.0001	0.0003
**Females 20Y (4X)**	N	145	139	143
	Median	38	35	18
	Difference, %		-23	-41
	χ^2		4.49	17.9
	p value		0.0342	<0.0001

Figure S1. Representative HPLC/MS spectra for AF. See methods and Table S1 for more details.

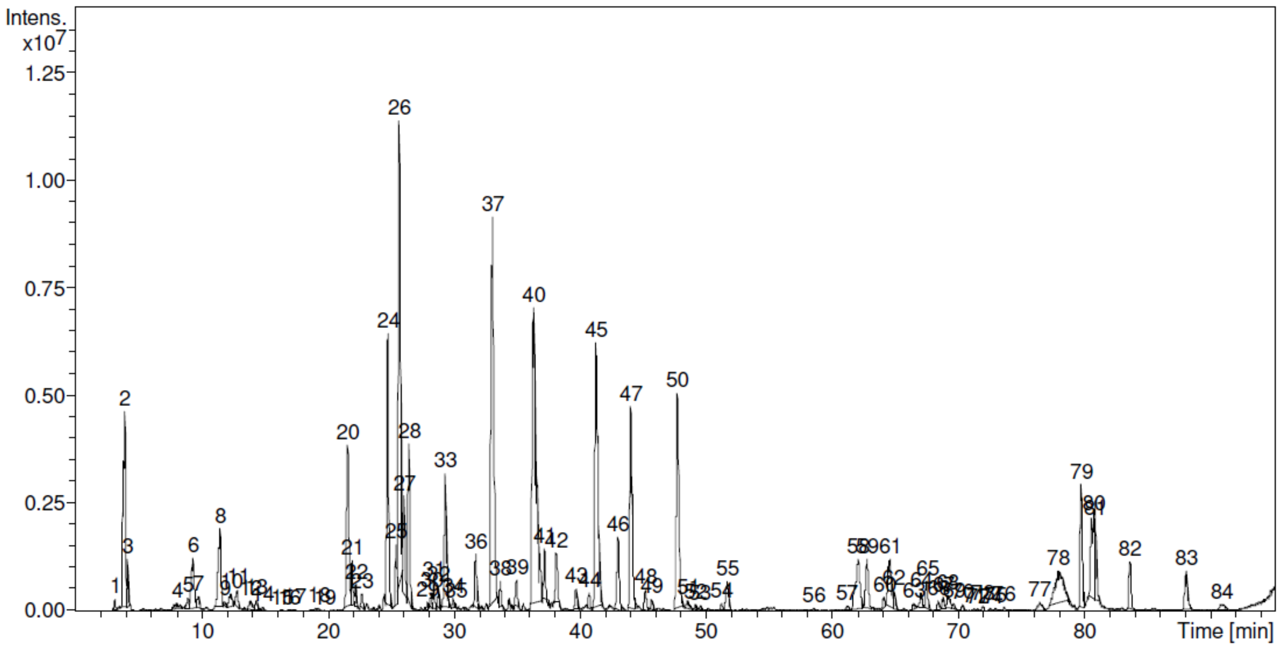

Strilbytska et al., Figure S1.
